# Association of immunological parameters with aortic dilatation in giant cell arteritis: a cross-sectional study

**DOI:** 10.1007/s00296-022-05186-1

**Published:** 2022-08-23

**Authors:** Philipp Jud, Nicolas Verheyen, Martin H. Stradner, Christian Dejaco, Dieter Szolar, René Thonhofer, Leyla Schweiger, Marianne Brodmann, Franz Hafner

**Affiliations:** 1 Division of Angiology, Department of Internal Medicine, Medical University of Graz, Auenbruggerplatz 15, 8036 Graz, Austria; 2 Division of Cardiology, Department of Internal Medicine, Medical University of Graz, Graz, Austria; 3 Division of Rheumatology, Department of Internal Medicine, Medical University of Graz, Graz, Austria; 4 Department of Rheumatology, Hospital of Brunico (SABES-ASDAA), Brunico, Italy; 5 Diagnostikum Graz Süd-West, Graz, Austria; 6 Department of Internal Medicine, State Hospital Muerzzuschlag, Muerzzuschlag, Austria

**Keywords:** Giant cell arteritis, Aorta, Inflammation, Interleukins, Cytokines

## Abstract

Aortic dilatation (AD) occurs in up to 30% of patients with giant cell arteritis (GCA). Reliable biomarkers for AD development, however, are still absent. The aim of this exploratory study was to evaluate whether immunological parameters are associated with the occurrence of AD in GCA. Cross-sectional study on 20 GCA patients with AD, 20 GCA patients without AD, and 20 non-GCA controls without AD measuring leukocytes, neutrophils, lymphocytes, C-reactive protein (CRP), erythrocyte sedimentation rate (ESR), serum amyloid A (SAA), interferon (IFN)-α, IFN-γ, IFN-γ-induced protein 10 (IP-10), interleukin (IL) 5, IL-8, IL-10, IL-17A, IL-18, IL-1 receptor antagonist, tumor necrosis factor (TNF)-α, platelet-derived growth factor (PDGF), L-selectin, P-selectin, and soluble intercellular adhesion molecule 1 (sICAM-1). AD was measured by aortic contrast-enhanced computed tomography and defined by enlargement of the aorta above population-based aortic diameters adjusted by age, gender, and body surface area. No significant differences were observed between GCA patients with AD and GCA patients without AD concerning levels of leukocytes, neutrophils, lymphocytes, CRP, ESR, SAA, IL-8, IL-18, PDGF, IP-10, selectins, and sICAM-1. Values of IFN-α, IFN-γ, IL-5, IL-10, IL-17A, IL-1 receptor antagonist, and TNF-α were all below the detection limits in more than 70% of subjects. Lymphocytes and CRP revealed positive correlations with the diameter of the thoracic descending aorta. Immunological parameters were not useful to conclude on the presence of AD in GCA. Further studies are required to test if CRP and lymphocytes may be useful to predict future development of AD in GCA.

## Introduction

Giant cell arteritis (GCA) is a large-vessel vasculitis commonly affecting supraaortic and cranial arteries [1]. The aorta is also commonly involved, especially the ascending and the descending thoracic aorta [2–4]. GCA patients are at an increased risk for the development of aortic damage, including aortic dilatation (AD)/aortic aneurysm, aortic rupture, and aortic dissection leading to higher mortality as compared to the general population and to GCA patients without AD [5–10]. Clinical parameters, including smoking, arterial hypertension, concomitant polymyalgia rheumatica (PMR) or male gender, have previously been associated with AD development [4, 5, 11]. In contrast, there are limited and controversial data about the possible linkage between immunological blood biomarkers and AD. Furthermore, only scarce data are available about underlying pathogenetic mechanisms for AD development in GCA. [5, 7, 12].

Since GCA represents an autoimmune vasculitis, there are implications that AD formation may be driven by inflammatory processes. White blood cell count (WBC) and acute-phase reactants, including C-reactive protein (CRP), erythrocyte sedimentation rate (ESR) and serum amyloid A (SAA), are commonly raised in GCA patients, and these factors have been demonstrated to predict a relapse of GCA [13–15]. Furthermore, some altered inflammatory parameters including ESR or specific lymphocytes subsets, seem to be associated with AD formation suggesting that AD development may be an evolving process initiated on the one hand in a phase of marked inflammation and may be maintained during the chronic phase of aortic remodelling [5, 7]. In addition, levels of interferon (IFN) γ, IFN-γ-induced protein 10 (IP-10), interleukin (IL) 8, IL-10, IL-17A, tumor necrosis factor (TNF) α, platelet-derived growth factor (PDGF), and soluble intercellular adhesion molecule 1 (sICAM-1) seem all to be altered in GCA patients as compared to controls. Most of these parameters were also linked to a higher disease activity in GCA [16–22]. Other cytokines such as IFN-α, IL-1 receptor antagonist (IL-1RA), IL-5, and L-selectin were found at comparable levels in GCA patients and controls [16, 18, 19, 22]; however, the potential involvement of IL-1RA, IL-5, and L-selectin in the development of arterial aneurysms has been suggested by animal models [23–25]. IL-18 and P-selectin may also have a potential effect on the development of aortic aneurysms, given that gene deletions of IL-18 and P-selectin attenuate the formation of aortic aneurysms in animals, probably by mitigation of the inflammatory response [26, 27].

While many of the above-named immunological parameters have been investigated in GCA, only acute-phase reactants were investigated as potential biomarkers for AD in GCA but there are no or limited data for IFN, IP-10, IL, TNF-α, PDGF, sICAM-1, and selectins [5, 7, 12]. Therefore, the aim of this study was to extend previous studies exploring the possible association of immunological parameters with the presence of AD in GCA patients.

## Materials and methods

### Study design and patient cohorts

This is an exploratory sub-analysis of a longitudinal study of GCA patients to investigate the development of AD in these patients. Full details about the study design and outcomes have been published previously [7]. In brief, patients with GCA diagnosed between 1993 and 2010 were identified by electronic chart review and were invited to participate in this study. Diagnosis of GCA was established clinically by the treating physician based on clinical parameters, laboratory data, imaging and/or biopsy results, while characteristic imaging and/or biopsy changes were mandatory for diagnosis of GCA. All subjects fulfilled retrospectively the modified ACR criteria proposed by Dejaco et al. [2, 14]. Only patients who had received a diagnosis of GCA for at least two years were included, assuming that AD is developing slowly and may thus evolve many years after disease onset [6]. Additionally, immunological analyses and CT measurements were performed in a phase of inactive GCA. Furthermore, anamnestic findings for other potential diseases leading to AD, including infections, connective tissue disorders, like Marfan syndrome, or congenital abnormalities, like Turner syndrome or bicuspid aortic valve, were checked at study inclusion, while no such findings or diseases were present. No subject had a disease relapse within a period of at least six months prior to the study visit. Exclusion criteria for GCA patients were active cancer, infections, active pregnancy or other types of vasculitis. Based on our search, we identified 144 patients with GCA, of whom 46 had AD according to population-based aortic diameters adjusted by age, gender, and body surface area [28]. Out of this group, we randomly selected 20 GCA patients with AD and 20 sex-matched GCA patients without AD. Twenty sex-matched non-GCA patients, who underwent thoracic and abdominal CT scans for control staging of inactive cancer or suspected pulmonary embolism were recruited as controls. Age-matching for GCA patients without AD and for non-GCA controls was not possible because the pool of GCA patients and non-GCA controls was not large enough to match for two (sex, age) variables. Healthy individuals were not included because it was considered unethical to expose healthy subjects to a CT scan to screen for AD, given the low prevalence of this disease in the general population [29]. Recent cancer progression, infections, and autoimmune diseases were exclusion criteria for the control group.

### Biochemical analyses

Fasting blood samples were obtained prior to CT for evaluation of immunological parameters including WBC (with neutrophils and lymphocytes), CRP, ESR, SAA, IL-5, IL-8, IL-10, IL-17A, IL-18, IL-1RA, TNF-α, IFN, IP-10, PDGF, selectins, and sICAM-1, as well as for evaluation of routine laboratory parameters, including hemoglobin, lipids, and kidney function. Routine laboratory parameters, WBC, CRP, and ESR were measured at a single central lab from sera and plasma of subjects. SAA, IL-5, IL-8, IL-10, IL-17A, IL-18, IL-1RA, TNF-α, IFN, IP-10, PDGF, selectins, and sICAM-1 were measured in sera, which were separated into aliquots after centrifugation and the respective aliquots were subsequently stored at − 20 °C until final analysis. Immunological parameters were measured in all 20 GCA patients with AD and in all 20 non-GCA controls. In one GCA patient without AD, analysis of the immunological parameters could not be conducted due to cracking of the blood sample during centrifugation. Therefore, immunological parameters were measured in 19 GCA patients without AD.

SAA was measured by particle-enhanced immunonephelometry on a Behring nephelometer system (BNA, CSL Behring, Vienna, Austria) according to the manufacturers protocol. IFN-α, IL-18, IL-1RA, IL-8, TNF-α (all in one ProcartaPlex panel, Invitrogen, Vienna, Austria), IFN-γ, IP-10, IL-10, IL-5, and IL-17A (all in one Platinum ProcartaPlex panel, Invitrogen) were analyzed by magnetic bead enhanced multiplex immunoassay according to the manufacturers protocol. In brief, sera were incubated with antibody coated magnetic beads. After addition of detection antibodies and Streptavidin–Phycoerythrin, samples were analyzed using a MAGPIX device (Merck Millipore, Darmstadt, Germany). L-selectin, P-selectin, and sICAM-1 (all ELISA kits by Invitrogen) and PDGF (Quantikine, R&D Systems, Minneapolis, USA) were analyzed using ELISA technique according to the manufacturer’s protocol.

### Computed tomography

Thoracic and abdominal aortic contrast-enhanced multi-detector CT was performed in all subjects in a supine position using a 64-slice scanner. Image acquisition was prospectively triggered in early diastole at 50% of the cardiac cycle and two acquisitions were conducted during end-inspiratory breath holds. Thoracic aortic diameters were measured at the ascending and descending thoracic aorta on axial slices at the level of the right pulmonary artery, and aortic diameters of the infrarenal abdominal aorta were taken on axial slices 5 cm above the aortic bifurcation. All measurements were traced manually from outside wall to outside wall of the aorta in anteroposterior planes and were performed by the same observer (D.S.). AD of the ascending aorta was defined by an aortic diameter of > 40 mm, AD of the thoracic descending aorta by a diameter of > 30 mm and AD of the infrarenal abdominal aorta by a diameter of > 20 mm [30, 31]. Furthermore, aortic diameters adjusted to age, gender, and body surface area above the 90^th^ percentile of the respective aortic region according to reported reference values by Rogers et al. [28] were also determined and defined as adjusted AD.

### Statistics

Continuous, non-normally, and normally distributed parameters were represented as median with interquartile range and means ± standard deviation (SD), respectively. Categorical parameters were expressed by frequency and percentages. Normal distribution was confirmed by the Kolmogorov–Smirnov test. The two-sided *t* test was used to compare mean values between groups in case of normally distributed data and the Mann–Whitney *U* test was used for non-normally distributed data. For categorical variables, the chi-squared test was utilized. Spearman’s or Pearson’s coefficients were applied in correlation analyses for non-normally or normally distributed variables, respectively. Statistical significance was assumed for *p* values < 0.05. Given the exploratory design no adjustment for multiple testing was applied. Statistical analyses were executed via SPSS version 26.0.

### Ethics and consent

The study was conducted according to the guidelines of the Declaration of Helsinki and approved by the Institutional Review Board (EK 23-312 ex 10/11). Informed consent was obtained from all subjects involved in the study.

## Results

GCA patients with and without AD did not differ concerning age, sex, and disease duration. Non-GCA controls were of similar age and sex as GCA patients. Eighteen (90%) GCA patients with AD and 19 (95%) GCA patients without AD received glucocorticoid therapy at the study visit (all with a daily dose < 7.5 mg). Two (10%) GCA patients with AD and two (10%) GCA patients without AD had received disease-modifying anti-rheumatic drugs (DMARDs) prior to the study visit. No control subject had a prior or current treatment with glucocorticoids or DMARDs. Patients’ characteristics are shown in Table 1.

**Table 1:** Patients’ characteristics at study inclusion

	GCA with AD (*n* = 20)	GCA without AD (*n* = 20)	Controls (*n* = 20)
Patients, *n* (%)			
Female	14 (70.0)	14 (70.0)	14 (70.0)
Male	6 (30.0)	6 (30.0)	6 (30.0)
Age (years)			
Mean (± SD)	69.9 (± 9.2)	72.5 (± 9.1)	72.0 (± 8.4)
Median (25th–75th percentile)	70.9 (63.4–77.6)	73.8 (63.1–77.6)	71.4 (65.7–80.2)
Disease types			
Predominately cranial GCA	5 (25.0)	7 (35.0)	–
Predominately extracranial GCA	15 (75.0)	13 (65.0)	–
Inactive cancer	–	–	10 (50.0)
Pulmonary embolism	–	–	10 (50.0)
Disease duration of GCA (years), mean (± SD)	4.6 (± 3.2)	5.1 (± 2.6)	–
Duration of prior glucocorticoid therapy (years), mean (± SD)	2.8 (± 2.8)	3.6 (± 3.0)	–
Hemoglobin (g/dl), median (25th–75th percentile)	13.3 (13.0–14.4)^#^	13.1 (12.3–14.2)^§^	11.8 (11.2–12.7)
Lipids (mg/dl), median (25th–75th percentile)			
LDL	125 (88–135)	135 (85–147)	99 (63–162)
HDL	60 (50–86)	66 (42–84)	56 (34–75)
Triglycerides	124 (82–163)	120 (87–183)	138 (93–196)
Lipoprotein (a)	9.5 (9.3–19.5)	15.1 (9.5–31.7)	9.5 (9.5–11.8)
Apolipoprotein A1	175 (150–198)	165 (138–195)	150 (102–175)
Apolipoprotein B	100 (77–112)	105 (81–113)	91 (70–135)
Kidney function, median (25th–75th percentile)			
eGFR (ml/min^−1^ 1.73 m^−2^)	69.88 (59.56–73.41)	63.84 (52.66–74.23)	80.71 (54.45–97.35)
Creatinine (mg/dl)	0.85 (0.79–1.04)	0.94 (0.79–1.22)	0.88 (0.63–1.11)
Previous history, *n* (%)			
Arterial hypertension	13 (65.0)	12 (60.0)	12 (60.0)
Diabetes mellitus	1 (5.0)	7 (35.0)*	5 (25.0)
Obesity	3 (15.0)	7 (35.0)	6 (30.0)
Hyperlipidemia	14 (70.0)	13 (65.0)	11 (55.0)
Smoking	8 (40.0)	7 (35.0)	6 (30.0)
Ex	3 (15.0)	3 (15.0)	4 (20.0)
Current	5 (25.0)	4 (20.0)	2 (10.0)
Coronary artery disease	2 (10.0)	5 (25.0)	5 (25.0)
Cerebrovascular disease	15 (75.0)^#^	10 (50.0)^§^	3 (15.0)
Upper extremity arterial disease	1 (5.0)	1 (5.0)	0 (0.0)
Lower extremity arterial disease	3 (15.0)	6 (30.0)	2 (10.0)
Renal artery disease	0 (0.0)	0 (0.0)	1 (5.0)
Mesenteric artery disease	0 (0.0)	0 (0.0)	0 (0.0)
BMI (kg/m^2^), mean (± SD)	26.7 (± 5.0)	27.3 (± 5.1)	26.3 (± 5.7)
Drug therapy, *n* (%)			
ACE inhibitors	5 (25.0)	7 (35.0)	6 (30.0)
Calcium antagonists	1 (5.0)	1 (5.0)	0 (0)
Beta blockers	6 (30.0)	10 (50.0)	4 (20.0)
Diuretics	2 (10.0)	4 (20.0)	5 (25.0)
Other antihypertensives	2 (10.0)	1 (5.0)	1 (5.0)
Insulin	0 (0.0)	2 (10.0)	1 (5.0)
Metformin	0 (0.0)	2 (10.0)	3 (15.0)
Statins	3 (15.0)	9 (45.0)	3 (15.0)
Antiplatelet therapy	8 (40.0)	17 (85.0)*^§^	7 (35.0)
Oral anticoagulation	3 (15.0)	2 (10.0)	4 (20.0)
Prior familial aortic aneurysms, *n* (%)	0 (0.0)	0 (0.0)	0 (0.0)
Aortic size (mm), mean (± SD)			
Thoracic ascending aorta	41.4 (± 3.6)*^#^	30.4 (± 2.8)	31.4 (± 1.9)
Thoracic descending aorta	29.9 (± 3.5)*^#^	23.8 (± 2.1)	23.2 (± 2.3)
Infrarenal abdominal aorta	19.0 (± 3.3)*^#^	16.5 (± 1.9)	16.7 (± 1.6)
Presence of AD, *n* (%)			
Thoracic ascending aorta			
> 40 mm	14 (70.0)*^#^	0 (0)	0 (0)
Adjusted diameter	17 (85.0)*^#^	0 (0)	0 (0)
Thoracic descending aorta			
> 30 mm	9 (45.0)*^#^	0 (0)	0 (0)
Adjusted diameter	12 (60.0)*^#^	0 (0)	0 (0)
Infrarenal abdominal aorta			
> 20 mm	3 (15.0)	0 (0)	0 (0)
Adjusted diameter	5 (25.0)*^#^	0 (0)	0 (0)

*ACE* angiotensin converting enzyme, *AD* aortic dilation, *BMI* body mass index, *eGFR* estimated glomerular filtration rate, *GCA* giant cell arteritis, *HDL* high-density lipoprotein, *LDL* low-density lipoprotein, *PMR* polymyalgia rheumatica

^*^p < 0.05 between group of GCA with AD and group of GCA without AD

^#^*p* < 0.05 between group of GCA with AD and controls

^§^*p* < 0.05 between group of GCA without AD and controls

Adjustment for multiple testing not performed due to exploratory character

Data on immunological blood biomarkers are depicted in Table 2. WBC, including neutrophils and lymphocytes, CRP, ESR, and SAA did not differ between GCA patients with AD, GCA patients without AD and non-GCA controls. Higher values of ESR were observed in non-GCA controls compared to GCA patients without AD (*p* = 0.038). IL-8, IL-18, PDGF, IP-10, L-selectin, P-selectin, and sICAM-1 did also not differ between GCA patients with AD, GCA patients without AD and non-GCA controls, while higher values of IP-10 were observed in the non-GCA control group as compared to GCA patients without AD (*p* = 0.015). The following markers were below the detection limit of the assay: IL-5 (detection limit 7.69 pg/ml) in 71.2% of all cases, IL-10 (detection limit 6.81 pg/ml) in 94.9%, IL-17A (detection limit 3.88 pg/ml) in 79.6%, IL-1RA (detection limit 18.14 pg/ml) in 89.8%, TNF-α (detection limit 4.84 pg/ml) in 94.9%, IFN-α (detection limit 0.26 pg/ml) in 94.9%, and IFN-γ (detection limit 2.00 pg/ml) in 86.4% of all subjects. Therefore, no conclusive statistical analyses were conducted for these parameters (Table 2).

**Table 2:** Inflammatory parameters between different cohorts

	GCA with AD (*n* = 20)	GCA without AD (*n* = 19)	Controls (*n* = 20)
WBC (10^9^/L), median (25th–75th percentile)	7.9 (5.8–9.5)	8.7 (7.2–9.98)	7.5 (5.67–11.1)
Neutrophils (10^9^/L)	5.1 (3.9–6.7)	5.4 (4.1–7.0)	4.5 (3.4–7.3)
Lymphocytes (10^9^/L)	1.6 (1.3–2.0)	1.9 (1.4–2.4)	1.5 (0.9–2.2)
CRP (mg/L), median (25th–75th percentile)	3.6 (2.6–7.7)	4.1 (1.6–12.5)	5 (1.0–32.8)
ESR (mm/h), median (25th–75th percentile)	13 (7–19)	8 (4–17)	14 (8–61)^§^
SAA (mg/L), median (25th–75th percentile)	4.6 (4.7–16.8)	7.8 (3.6–17.75)	10.0 (3.6–28.8)
Interleukins (pg/mL), median (25th–75th percentile)			
IL-5	–^†^	–^†^	–^†^
IL-8	8.38 (6.99–15.54)	9.45 (6.96–12.10)	10.99 (6.37–24.33)
IL-10	–^†^	–^†^	–^†^
IL-17A	–^†^	–^†^	–^†^
IL-18	65.28 (44.79–112.33)	76.71 (49.12–114.26)	86.54 (49.91–124.35)
IL-1RA	–^†^	–^†^	–^†^
TNF-α (pg/mL), median (25th–75th percentile)	–^†^	–^†^	–^†^
PDGF (pg/mL), median (25th–75th percentile)	13,790.7 (10,083.6–17,493.5)	12,138.3 (11,301.7–14,908.8)	10,464.1 (6830.4–15,996.2)
Interferons (pg/mL), median (25th–75th percentile)			
IFN-α	–^†^	–^†^	–^†^
IFN-γ	–^†^	–^†^	–^†^
IP-10 (pg/mL), median (25th–75th percentile)	119.57 (71.98–161.32)	92.05 (67.13–142.98)	168.31 (102.48–224.68)^§^
Selectins (ng/mL), median (25th–75th percentile)			
L-selectin	2679.3 (2225.0–2855.7)	2554.2 (2280.6–2897.3)	2425.7 (1971.8–2891.9)
P-selectin	132.2 (117.8–164.4)	133.3 (117.6–146.3)	122.8 (90.1–151.6)
sICAM-1 (ng/mL), median (25th–75th percentile)	488.9 (432.8–546.0)	504.0 (409.7–541.3)	509.4 (432.8–615.5)

*CRP* C-reactive protein, *ESR* erythrocyte sedimentation rate, *IFN* interferon, *IP-10* interferon-gamma-induced protein 10, *IL* interleukin, *IL-1RA* interleukin-1 receptor antagonist, *PDGF* platelet-derived growth factor, *SAA* serum amyloid A, *sICAM-1* soluble intercellular adhesion molecule 1, *TNF* tumor necrosis factor, *WBC* white blood cells

^*^p < 0.05 between group of GCA with AD and group of GCA without AD

^#^*p* < 0.05 between group of GCA with AD and controls

^§^*p* < 0.05 between group of GCA without AD and controls

^†^Statistical analysis was not conducted as the values of the respective parameter were below the detection limit in ≥ 70%

Adjustment for multiple testing not performed due to exploratory character

Within the group of GCA patients with AD, moderate correlations were found between the diameter of the thoracic descending aorta and lymphocyte counts (*r* = 0.504, *p* = 0.023) or CRP (*r* = 0.519, *p* = 0.019). The other immunological parameters did not reveal such an association (Table 3).

**Table 3:** Correlation matrix of aortic diameters and immunological parameters within GCA patients with AD unadjusted for multiple testing

		WBC	Neutrophils	Lymphocytes	CRP	ESR	SAA	IL-8	IL-18	IP-10	PDGF	L-selectin	P-selectin	sICAM-1
Diameter ascending aorta	*r*	− 0.184	− 0.139	− 0.120	− 0.444	− 0.195	− 0.152	0.208	− 0.015	0.354	− 0.443	0.199	− 0.166	0.079
	*p*	0.438	0.560	0.616	0.050	0.409	0.522	0.379	0.949	0.126	0.051	0.400	0.485	0.739
	*n*	20	20	20	20	20	20	20	20	20	20	20	20	20
Diameter thoracic descending aorta	*r*	0.093	− 0.098	**0.504**	**0.519**	0.256	0.044	− 0.279	− 0.197	− 0.293	0.230	− 0.008	− 0.204	− 0.379
	*p*	0.697	0.681	**0.023**	**0.019**	0.275	0.854	0.234	0.404	0.210	0.329	0.975	0.387	0.099
	*n*	20	20	20	20	20	20	20	20	20	20	20	20	20
Diameter infrarenal abdominal aorta	*r*	0.087	0.129	− 0.013	0.365	− 0.139	− 0.214	0.061	− 0.041	− 0.171	0.067	− 0.039	− 0.184	− 0.112
	*p*	0.714	0.589	0.958	0.114	0.558	0.365	0.797	0.864	0.471	0.780	0.870	0.436	0.639
	*n*	20	20	20	20	20	20	20	20	20	20	20	20	20

Significant values are in bold

*CRP* C-reactive protein, *ESR* erythrocyte sedimentation rate, *IP-10* interferon-gamma-induced protein 10, *IL* interleukin, *PDGF* platelet-derived growth factor, *SAA* serum amyloid A, *sICAM-1* soluble intercellular adhesion molecule 1, *WBC* white blood cells

Adjustment for multiple testing not performed due to exploratory character

## Discussion

In the present study, we did not observe any association of the immunological blood biomarkers tested with the presence of AD in patients with GCA. Lymphocyte counts and CRP moderately correlated with the diameter of the thoracic descending aorta, however, both parameters did not differ between GCA patients with and without AD. We acknowledge that the study population of 20 subjects per group was low and that the number of immunological parameters tested was high. Therefore, we cannot exclude a type 1 error resulting in an incidental association of CRP and lymphocytes with AD. Further studies with a larger sample size are, therefore, needed to validate our results. Nevertheless, we believe that these parameters play only a minor role in the development of AD.

There is limited and controversial data about the possible value of routine blood parameters as potential markers of AD formation in GCA. Gonzalez-Gay et al. [5] suggested that a distinct inflammatory response represented by ESR > 100 mm/h and/or hemoglobin < 11 g/dl and/or platelet count > 450,000/mm^3^ at disease onset predicted AD formation in GCA. However, another study did not confirm this observation given that increased acute-phase reactants at disease onset, like ESR, CRP or fibrinogen, were not associated with the development of AD [7]. In a third study, GCA patients with AD revealed lower ESR levels after a median follow-up of 5.4 years than GCA patients without AD [12]. In that study, no differences between GCA with AD and without AD were observed concerning CRP, IL-6, IL-18 and TNF-α, which is in line with our results. Whether persisting low-grade inflammation or distinct alterations of specific cytokines and inflammatory parameters at disease onset or in a phase of inflammation may contribute to the development of AD in GCA is unclear as it is the role of immunosuppressive agents in preventing damage of the aortic wall. Glucocorticoids, which are the main therapeutic agent for GCA, rapidly reduce the systemic inflammatory response but were unable to abrogate inflammation in the arterial wall in a murine model of GCA [32]. Furthermore, acute-phase reactants, like CRP or SAA, were associated with aortic injury, including aortic aneurysm and aortic dissection, in atherosclerotic disease [33–35]. Whether these results can be extrapolated to GCA patients is unclear, but clinical observations confirm that AD developed even in patients in full clinical remission and under continuous glucocorticoid therapy [7, 8, 11]. It can only be speculated whether the initial inflammatory response may cause an injury of the aortic wall eventually leading to remodelling and AD formation, which is not or little influenced by immunosuppressive agents anymore. Our results are in favour of this hypothesis given that we did not detect an inflammatory signature in our GCA group with AD, and that all of the included GCA patients had received initial glucocorticoid therapy at disease onset which was continued for at least 6 months.

We do certainly not know at what time point AD occurred or when it progressed. For this purpose, another study design like a multi-annual observational study with repeated radiological aortic measurement is necessary. Therefore, we cannot exclude that aortic damage was promoted in a phase of inflammation which was then followed by a period of immunological quiescence. On the other hand, only two patients (10%) had advanced aortic aneurysms and also the mean size of aortic aneurysms in the group of GCA patients with AD was relatively low (41.4 ± 3.6 mm for the ascending aorta; 29.9 ± 3.5 mm for the thoracic descending aorta; 19.0 ± 3.3 mm for the infrarenal abdominal aorta). Therefore, we can assume that the majority of patients either had mild AD or were captured at an early stage of AD. Our GCA cohorts had mean disease durations of 4.6 and 5.1 years, respectively, which are comparable to previous studies investigating AD in GCA while it must be noted that GCA is a chronic disease and that large aortic aneurysms, especially thoracic aneurysms, may occur more frequently after a median disease duration of 10.9 years [5, 6, 12].

Even though we did not observe differences between GCA patients with and without AD concerning the cytokines tested, several of blood markers have been linked with the development of aortic injury: Elevated levels of IL-5, IL-6, IL-17A, IFN-γ, TNF-α, and PDGF were all observed in the blood of patients with aortic aneurysms and/or aortic dissection [36, 37]. Besides, animal models revealed that deficiencies of P-selectin attenuate and those of IP-10 enhance abdominal aortic aneurysm formation [27, 38]. Most of these studies, however, evaluated the role of cytokines in atherosclerotic abdominal aortic aneurysms while data on aortic aneurysms related to immune-mediated diseases, which mostly affect the thoracic aorta, are scarce.

The thoracic and abdominal aorta feature differences in histology and expression of chemokine receptors resulting in a different cytokine profile after stimulation [39, 40]. It can, therefore, be assumed that the pathogenesis of atherosclerotic and inflammatory AD follows different mechanisms which in part determined by the local milieu with resident stroma and immune cells as well as chemokine receptors [7, 41, 42]. The different susceptibility of different aortic regions to inflammatory stimuli, the heterogeneous cytokine expression in aortic aneurysms, and the fact that almost each GCA patient have received glucocorticoid therapy at the study visit may explain the findings that our control cohort had normal aortic diameter but higher levels of several cytokines, including IP-10, sICAM-1 or IL-8, and that there were no differences of the measured cytokines between the two GCA subgroups.

One limitation of our study is the exploratory design without adjustment for multiple testing which only allows for hypothesis generation. The small sample size and the use of disease controls are not ideal to investigate the association of blood biomarkers with AD and may have potentially biased the results. On the other hand, this study was a sub-analysis of a previous study and was intended as a pilot study investigating the linkage of several selected immunological parameters to AD in GCA, which have not been evaluated before to the best of our knowledge [7]. Another limitation was that no follow-up computed tomography (CT) measurements of the aorta were performed. Therefore, we cannot make any conclusion about the possible predictive value of immunological blood biomarkers for the future development or progression of AD. Further limitation was the impossibility to match the three groups for age, given that age is an established risk factor for the development of AD. However, the mean age of the groups was nevertheless similar so that an impact of this factor on our results seems to be negligible. The strengths of our study are a consistent CT measurement to detect AD, definitions of AD with a fixed cut-off diameter and with adjustments to age, gender and body surface area. Another strength of this study is that several immunological parameters, including IP-10, IL-8, PDGF, sICAM-1, and selectins, have not been tested previously as potential biomarkers for the presence of AD in GCA.

The majority of our GCA patients had AD in the thoracic aorta, while abdominal AD occurred in 5 (25%) patients only. The relevance of the investigated blood biomarkers for predicting the development or explaining the pathogenesis of abdominal AD in GCA patients is, therefore, limited and requires further clarification.

## Conclusion

In conclusion, immunological parameters are probably not useful to conclude on the presence of AD in GCA. Lymphocyte and CRP levels may be linked with the diameter of the thoracic descending aorta in GCA patients with AD, but this warrants confirmation in prospective and larger cohorts. Further studies with long-term follow-up data are needed to clarify the possible role of immunological parameters as predictors for the future development of AD in GCA.

## Abbreviations

AD: Aortic dilatation
CRP: C-reactive protein
CT: Computed tomography
DMARD: Disease-modifying anti-rheumatic drug
ESR: Erythrocyte sedimentation rate
GCA: Giant cell arteritis
IFN: Interferon
IP-10: Interferon-gamma-induced protein 10
IL: Interleukin
IL-1RA: Interleukin-1 receptor antagonist
PDGF: Platelet-derived growth factor
PMR: Polymyalgia rheumatica
SAA: Serum amyloid A
SD: Standard deviation
sICAM-1: Soluble intercellular adhesion molecule 1
TNF: Tumor necrosis factor
WBC: White blood cell count
